# Structural insights and functional implications of inter-individual variability in β_2_-adrenergic receptor

**DOI:** 10.1038/srep24379

**Published:** 2016-04-14

**Authors:** Aditi Tandale, Manali Joshi, Durba Sengupta

**Affiliations:** 1 CSIR-National Chemical Laboratory, Dr. Homi Bhabha Road, Pune 411008, India; 2 Bioinformatics Centre, S.P. Pune University, Ganeshkhind Road, Pune 411007, India

## Abstract

The human β_2_-adrenergic receptor (β_2_AR) belongs to the G protein-coupled receptor (GPCR) family and due to its central role in bronchodilation, is an important drug target. The inter-individual variability in β_2_AR has been implicated in disease susceptibility and differential drug response. In this work, we identified nine potentially deleterious non-synonymous single nucleotide polymorphisms (nsSNPs) using a consensus approach. The deleterious nsSNPs were found to cluster near the ligand binding site and towards the G-protein binding site. To assess their molecular level effects, we built structural models of these receptors and performed atomistic molecular dynamics simulations. Most notably, in the Phe290Ser variant we observed the rotameric flip of Trp286^6.48^, a putative activation switch that has not been reported in β_2_AR thus far. In contrast, the variant Met82Lys was found to be the most detrimental to epinephrine binding. Additionally, a few of the nsSNPs were seen to cause perturbations to the lipid bilayer, while a few lead to differences at the G-protein coupling site. We are thus able to classify the variants as ranging from activating to damaging, prioritising them for experimental studies.

The human β_2_-adrenergic receptor (β_2_AR) is a seven transmembrane protein of the G protein-coupled receptor (GPCR) family that is encoded by the ADRB2 gene^1^. The β_2_AR is predominantly expressed in smooth muscles throughout the body, most abundantly in the lungs^2^. The binding of endogenous catecholamine agonists, such as epinephrine, triggers the activation of adenylyl cyclase via the Gs protein which leads to muscle relaxation^3^. The β_2_AR is expressed largely in smooth airway muscles of the lung where it plays a critical role in respiration. Drugs targeting β_2_AR are prescribed in asthmatic conditions that lead to receptor activation, causing bronchodilation^4^. β_2_AR agonists are also used for uterine smooth muscle relaxation to prevent preterm labour^5^,^6^. The expression of β_2_AR on adipocytes has been seen to be correlated with lipolysis having implications on obesity and cardiovascular function^7^,^8^. Further, β_2_AR is associated with insulin signalling and is purported to have a role in type II diabetes^9^,^10^. In addition, non-selective antagonists of β_1_-adrenergic receptor (β_1_AR), prescribed for cardiovascular diseases are known to inactivate the β_2_AR at high concentrations causing shortness of breath as a side effect^11^. The β_2_AR thus plays a pivotal role in the regulation of the cardiac, pulmonary, vascular and endocrine systems and hence the inter-individual variability in the receptor will have effects on various diseases.

Several single nucleotide polymorphisms (SNPs) have been described for β_2_AR that are associated with disease susceptibility and a differential therapeutic response^12^. In particular, the variants at positions 16, 27 and 164 have been correlated with a risk for asthma, diabetes and obesity as well as a differential therapeutic response to asthma and cardiovascular drugs^13^. However the correlations between these SNPs and disease risk or therapeutic response differ across studies and remain to be replicated in larger studies. The advent of massive genome sequencing projects such as the 1000 genome project have enabled the detection of inter-individual genome variability, potentially uncovering rare and low frequency variants that have not been captured by clinical studies thus far^14^. These polymorphisms may alter receptor dynamics affecting ligand binding and G-protein coupling through local and long-range effects in subtle but important ways^15^. It is thus critical to understand the molecular level details of how these SNPs could affect β_2_AR function and their implications in physiology.

The β_2_AR has been well characterized structurally with multiple crystal structures of the receptor in native and various ligand bound forms^16^. The ligands of β_2_AR are seen to bind at a buried site between transmembrane (TM) helices 3, 5, 6 and 7 with the residues Asp113, Tyr316 and Asn312 and Ser203, Ser207 and Asn293 defining the topology of the binding site^17^. A seminal study was able to predict the dominant binding pathway for β_2_AR ligands using unbiased molecular dynamics simulations achieving poses matching those determined crystallographically^18^. Insights into the active structure of β_2_AR were revealed by a crystal structure of the G-protein coupled to β_2_AR and agonist. This structure reveals that the Gα protein binds at a cleft between TM helix 5 and 6, induced by the large outward movement of TM helix 6 and helical extension of TM helix 5^19^. Specifically, Arg131 and Phe139 form critical contacts with the α subunit of the G-protein^19^. Interestingly, the β_2_AR can adopt the active state only in the presence of agonist and G-protein, as shown by a combined crystallography and molecular dynamics study^20^. Further, electron microscopy studies have gleaned insights into the association of β_2_AR with β-arrestin^21^. β-arrestin is observed to interact by a biphasic mechanism, the first at the C-terminal domain of β_2_AR followed by weak interactions with the receptor core.

Dynamic studies combining NMR with molecular dynamics (MD) simulations have provided insights into the activation mechanism of the receptor^22^,^23^,^24^,^25^. A loose allosteric network responsible for activation couples the ligand binding pocket to the intracellular G-protein binding site via a connector site (Ile121 and Phe282)^22^,^26^. The stabilization of the outward pointing TM helix 6 is thought to be concerted with the flipping of the sidechain of Trp286 (residue 6.48 of GPCRs as per the Ballesteros Weinstein numbering), called the rotamer toggle switch model (recently called transmission switch model) for receptor activation^27^. This switch however has only been observed in experimental studies of rhodopsin^28^,^29^. Recently, the activation of class A GPCRs has also been linked to the formation of a continuous water channel that is facilitated by the flipping of Tyr326 (residue 7.53 of GPCRs as per the Ballesteros Weinstein numbering) and the opening of an adjacent hydrophobic layer of amino acids^30^. Thus, the β_2_AR exhibits high conformational plasticity, and the dynamics between multiple states are linked to the activation mechanism^31^. Allosteric modulation by phospholipids have been observed experimentally^32^ and predicted by simulations^33^. This well characterized activation mechanism of the wildtype β_2_AR provides a benchmark to compare the dynamics of the natural variants.

In this work we characterize non synonymous single nucleotide polymorphisms (nsSNPs) in the β_2_AR by analysing molecular level differences that have functional implications. Towards the same we compiled naturally occurring nsSNPs of β_2_AR and predicted deleterious mutations using the consensus predictor, PredictSNP^34^. Subsequently all putative deleterious nsSNPs were structurally modelled and simulated in atomistic detail, totalling to 10.4 μs of simulation time. We identified structural differences in the receptor and membrane perturbations induced by these variants. Our results suggest that some nsSNPs affect ligand and G-protein binding while others cause large membrane perturbations. Surprisingly, one of the nsSNPs predicted to be deleterious appears to be activating. Our work suggests *in vitro* testing of these polymorphisms, followed by clinical studies that would reveal their role in health and disease.

## Results

In this work, deleterious nsSNPs of β_2_AR were identified and mapped structurally to gain molecular insights into their effects on function. The dbSNP was mined to compile a list of nsSNPs of the ADRB2 that was further refined based on their deleterious nature. These deleterious variants were modelled and simulated to identify their structural variations.

### Prediction and structural modelling of deleterious nsSNPs in β_2_AR

**Figure 1:**
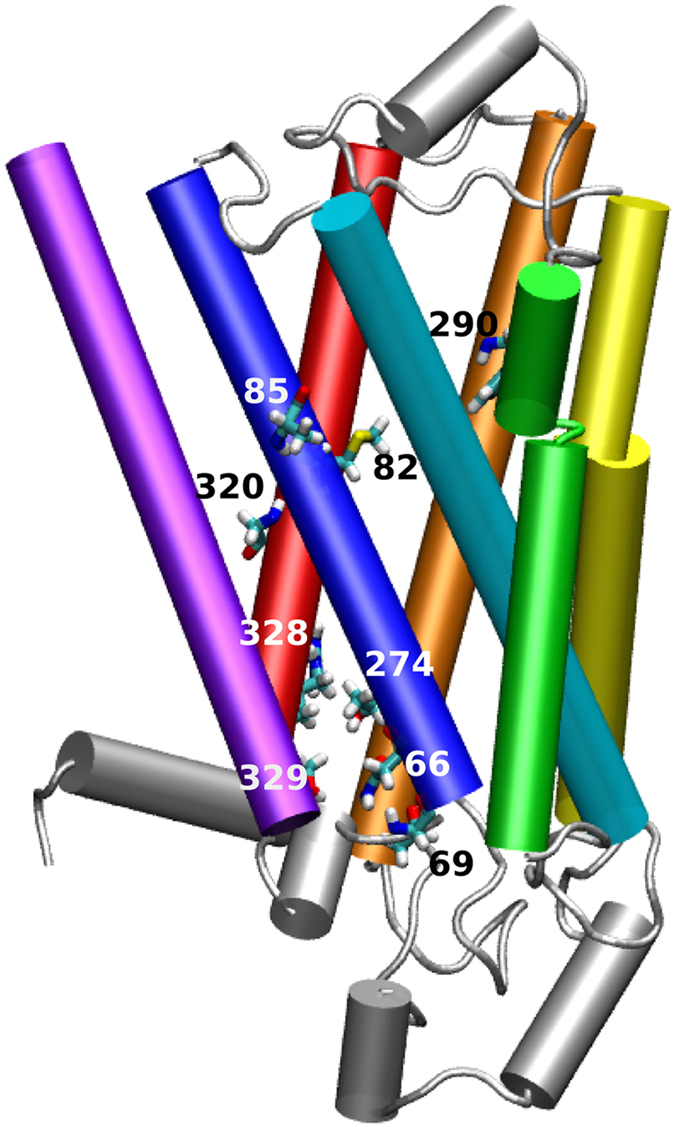
A schematic representation of β_2_AR and its variants. The transmembrane helices of β_2_AR are represented as cylinders and coloured. The intracellular and extracellular loops and helix 8 are coloured in silver. The positions harbouring the deleterious mutations are rendered in licorice representation and are labelled.

Mining the dbSNP revealed that the ADRB2 gene had a total of 290 SNPs of which 41 were nsSNPs (Supplementary Table S1). To determine which of these nsSNPs were possibly deleterious we used a consensus prediction tool, PredictSNP that has been recently suggested to provide an accurate and robust prediction^34^. The individual tools comprising PredictSNP give predictions based on evolutionary, physico-chemical or structural characteristics and are supplemented by experimental annotations from Protein Mutant and UniProtKB databases. Based on individual predictions, PredictSNP classifies SNPs as being neutral or deleterious. Table 1 lists nine of the 41 nsSNPs that were predicted to be deleterious along with their final score. The predictions were confirmed to be detrimental using two other tools namely, Meta-SNP^35^ and PON-P2^36^. The nine nsSNPs were mapped to the β_2_AR structure as described in the methods section (Fig. 1). All the nine nsSNPs were found to be located on TM helices, or at their termini. The nsSNPs are observed to cluster at two general areas, around the ligand binding site and near the G-protein coupling site. The nsSNPs at positions 82, 85, 290 and 320 were found to be near the ligand binding site while the nsSNPs at positions 66, 69, 274, 328 and 329 were found to be clustered near the G-protein coupling site. Further, three of the nine variants, at positions 85, 274 and 320 were observed to point towards the membrane, while the rest were observed to point toward the lumen of the receptor.

### Structural differences in deleterious nsSNPs and their effects on ligand binding

**Figure 2:**
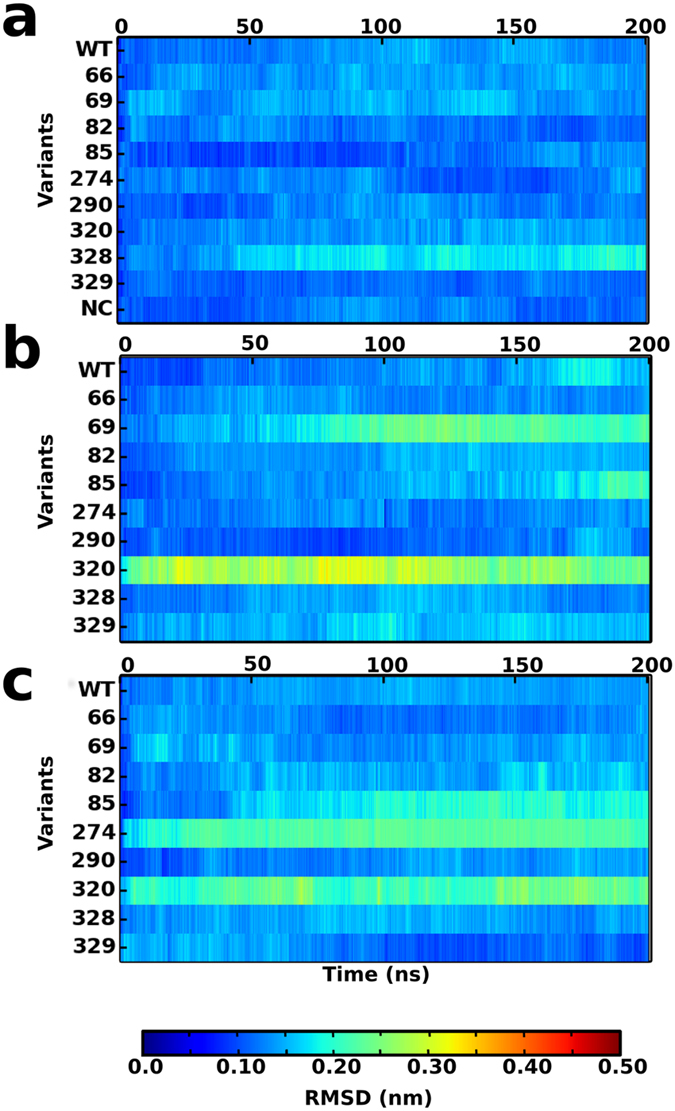
Structural characterization of the mutant receptors of β_2_AR. RMSD of the transmembrane helices (backbone) for (**a**) simulation set 1 (**b**) set 2 and (**c**) set 3 with respect to 2RH1 crystal structure. The RMSD for the variant Gln179Glu has been plotted for one set and is labelled as NC (Negative Control).

In order to gain insights into how the nsSNPs might affect dynamics of the receptor, atomistic simulations of 200 ns were performed in triplicate for the wildtype and nine variants, totalling to 6 μs. In addition, the nsSNP Gln179Glu classified as neutral (with a confidence of 83%) was modelled and simulated for the same time scale as the others and was treated as a negative control. Differences in the structural characteristics of the variants were investigated. Since all the deleterious nsSNPs were located in the TM region, we analysed their effect on the stability of the helices by plotting their RMSD (Fig. 2). As expected the wildtype receptor shows a very low RMSD (<0.2 nm) suggesting highly stable TM helices. The variation at positions 69, 85, 274 and 320 display increased fluctuations in the TM helices. The remaining variants including the negative control had a stable RMSD over the length of the simulation.

**Figure 3:**
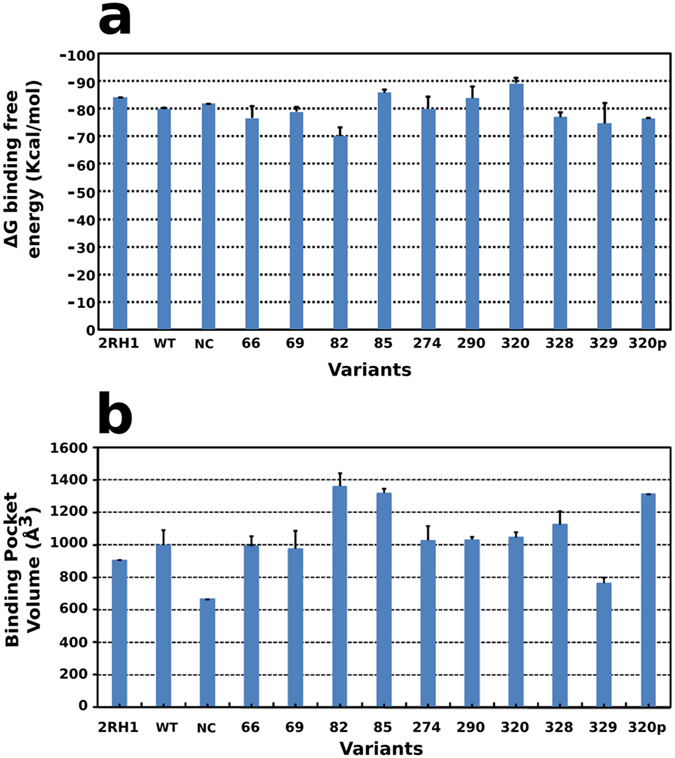
Characterization of ligand binding. (**a**) The binding free energy of R-epinephrine averaged over simulation triplicates. (**b**) Binding pocket volume averaged over simulation triplicates.

To analyse whether the variants affect binding of R-epinephrine, its natural ligand, the binding free energy of epinephrine was calculated as discussed in the methods section. It should be noted that the binding free energies are calculated using an implicit membrane model and only the relative ranking is meaningful^37^. The binding of epinephrine was favourable in the wildtype receptor, as compared to the variants (Fig. 3a). Additionally, the binding free energy of epinephrine to the wildtype structures and the negative control obtained after 200 ns of simulation were similar. Marginal differences were observed in the binding free energies of the mutants at positions 320 and 328, as compared to the wildtype receptor. The variant at the 82^nd^ position displayed the least favourable binding free energy in all three sets. Further, the volume of the binding site was calculated from the non-occluded space in the lumen created by residues defining the binding site (Fig. 3b). Interestingly, the variant at position 82 displays the largest binding pocket volume and the least favourable binding free energy.

### Phe290Ser demonstrates the rotamer toggle switch for receptor activation

**Figure 4:**
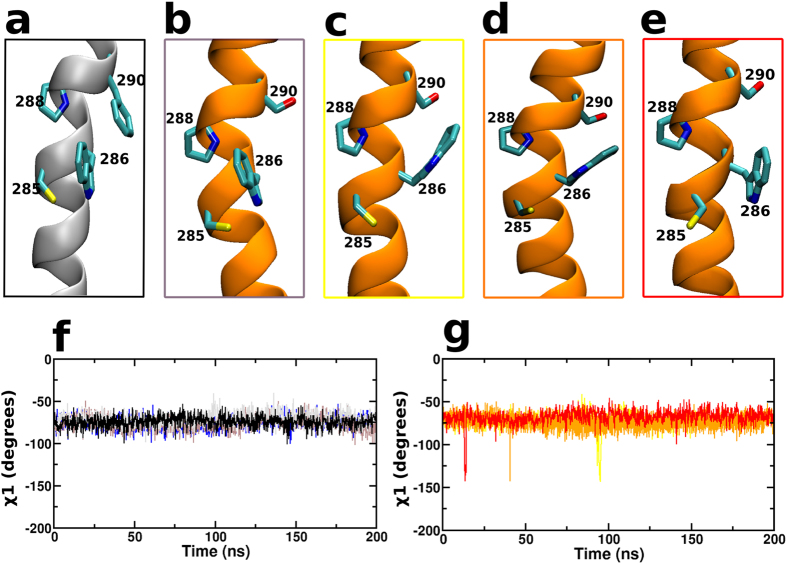
Rotamer toggle switch in Phe290Ser. (**a**) TM helix 6 of β_2_AR crystal structure (2RH1) with the key residues, including the wildtype residue Phe 290, labelled and rendered in licorice. Representation of TM helix 6 of the Phe290Ser variant at the start (**b**) and the end of the simulation sets (**c**–**e**) for the three simulation sets. The key residues, including the variant residue Ser 290, are labelled and rendered in licorice representation. A plot of the dihedral angle, χ^1^ of Trp286 along the course of the simulation for (**f**) the wild-type (black, grey, brown) and negative control (blue) and (**g**) the Phe290Ser simulation sets (red, orange, yellow).

The tryptophan at position 286^6.48^ is conserved in GPCRs and is located at the bottom of the binding site. This residue has been suggested to function as a rotamer toggle switch (also called the transmission switch) in the GPCR activation mechanism. The rotamer toggle switch involves a *gauche* (inactive) to *trans* (active) transition of the χ^1^ angle of Trp^6.48^ facilitated by a similar flip of the neighbouring Phe290^6.52^. This model was based on experiments and simulations of rhodopsin and adenosine_2A_ receptor^28^,^29^,^38^,^39^, respectively. In the crystal structures of β_2_AR (Fig. 4a) the Trp286^6.48^ is seen to adopt a rotamer associated with the inactive state (*gauche*) that is sterically constrained by Phe290^6.52^. The flip of this sidechain has not been reported in crystal structures or MD simulations of the wildtype β_2_AR. The nsSNP at position 290 involves the mutation of a phenylalanine to serine, a non-aromatic amino acid, this led us to investigate its effect on the rotameric flip of Trp286^6.48^. Towards the same we monitored the χ^1^ angle of Trp286^6.48^ for the wildtype, negative control and the Phe290Ser variants (Fig. 4). The plot indicates that there is a transient flip of the χ^1^ of Trp286^6.48^ from the *gauche* to *trans* conformation in all three sets of the simulation. This is visually represented in Fig. 4c–e. The χ^1^ angle in the wildtype receptor remains constant and the Phe290 maintains a close aromatic packing preventing the flip of Trp286^6.48^. The flip is absent in the other eight variants (Supplementary Fig. S1). Since the rotamer flip is proposed to be one of the first steps of receptor activation, the results suggest that the Phe290Ser variant can be activating. However, microsecond timescale conformational changes reflecting activation were not observed.

### Membrane facing nsSNPs cause perturbations in the bilayer

**Figure 5:**
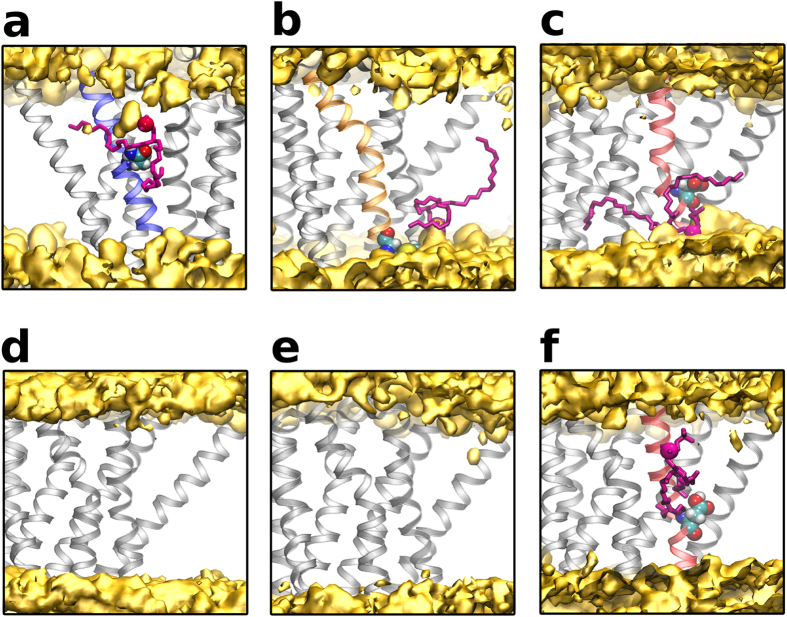
Membrane perturbations in Ala85Gln, Thr274Met and Gly320Asp. Schematic representation of average lipid head group (phosphate atom) position of POPC in the vicinity of the receptor for (**a**) Ala85Gln, (**b**) Thr274Met (**c**) Gly320Asp (**d**) wildtype (**e**) negative control (**f**) protonated Gly320Asp. The values in (**a**) to (**d**) are averages over three simulation sets. The receptor is shown in grey helical representation while the TM helix harbouring the mutation is coloured as per Fig. 1. The perturbed lipid is rendered in licorice and coloured magenta, while the average head group density is coloured yellow.

Three nsSNPs Ala85Gln, Thr274Met and Gly320Asp were observed to point towards the membrane, out of which the first and third are located towards the binding site while the second is close to the intracellular region. Since the mutation at the 85^th^ and 320^th^ position results in the introduction of a polar and charged residue respectively in the hydrophobic core of the membrane, we investigated whether the variants caused perturbations in the bilayer. The perturbations were characterized in terms of average lipid headgroup position in the vicinity of the mutation and is shown as a schematic representation in Fig. 5. A visual analysis showed that in the wildtype receptor and negative control the average position of the lipid headgroup (phosphate atom) was not perturbed (Fig. 5d,e). On the other hand, in the Ala85Gln variant, the glutamine sidechain had a close interaction with the polar groups of a POPC molecule (Fig. 5a), pulling it inward causing the perturbation of the surrounding lipids. At the equivalent position in the wildtype receptor, the alanine sidechain interacts with the lipid tails. In the Thr274Met mutant, the methionine sidechain interacts with the choline head group of POPC (Fig. 5b) whereas, in the wildtype receptor the hydroxyl group of threonine forms an intermittent hydrogen bond with the backbone carbonyl of Lys270. In the variant Gly320Asp the charged aspartate residue located at the middle of the receptor causes large bilayer perturbations. POPC molecules from either the intracellular or the extracellular leaflet are pulled in towards Asp320 (Fig. 5c). This variant has an increased number of water molecules in the vicinity of the variant residue as compared to the wildtype receptor (Supplementary Fig. S2). As a control, simulations were performed with a protonated Asp. A close interaction with a phospholipid was observed, although the surrounding lipids were less perturbed. Interestingly, all three variants displayed high RMSD (Fig. 2).

To analyse whether the lipid association causes bilayer thickness perturbations, the bilayer thickness profile was calculated (Supplementary Fig. S3). The bilayer thickness profile around the wildtype receptor shows membrane thickening (indicated by red in the figure) at the groove formed by TM helix 1 and 7 and marginal thinning (blue) at the other sites in the vicinity of the receptor, consistent with previous reports^40^,^41^. In contrast, the membrane thickening at the groove between TM helix 1 and 7 was reduced in all three mutants (Supplementary Fig. S3). Additionally, the Ala85Gln variant showed reduced bilayer thickness around TM helix 1 and 2 that were seen to cause larger perturbations in the whole bilayer. Similarly variants at position 274 and 320 caused large bilayer thinning in the vicinity of the receptor. Thus, large membrane perturbations are seen in the vicinity of the receptor in all three variants.

### Met82Lys causes rearrangements and electrostatic changes in the binding site

**Figure 6:**
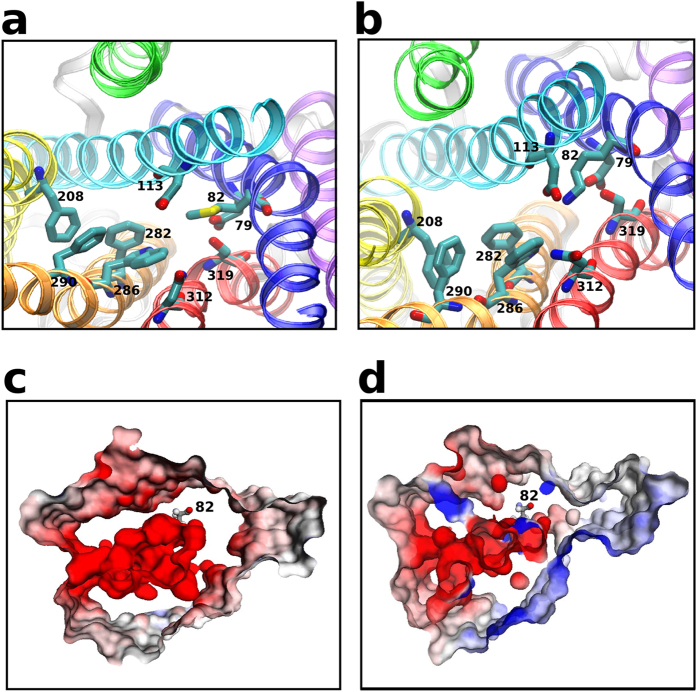
Characterisation of the ligand binding site of the Met82Lys variant. A schematic representation of the binding site of the (**a**) wildtype receptor and (**b**) Met82Lys variant. The TM helices are shown as ribbons and coloured as in Fig. 1. The important residues in the binding site, and the variant residue are shown as licorice. Electrostatic potential maps of the binding site of (**c**) wild type set 1 and (**d**) Met82Lys variant set 1.

The binding site of the wildtype β_2_AR has been reported to have a negative electrostatic potential facilitating the binding of positively charged catecholamine ligands (Fig. 6). The variant Met82Lys is situated at the periphery of the binding site (Fig. 6 a,b) and introduces a positive charge leading to electrostatic changes in its vicinity(Fig. 6 c,d). The altered electrostatics affects binding of epinephrine and is reflected in the poor binding free energy as described earlier (Fig. 3a). Lys82 is observed to form hydrogen bonds with the sidechain of residues Asp113, Asp79, Trp286 or Ser319 leading to rearrangements in the binding site. Specifically, the distance between the Trp286 and Tyr316 increases in the mutant due to the intermittent hydrogen bonding of Trp286 with Lys82 (Supplementary Fig. S4). This leads to a rearrangement in the aromatic core at the bottom of the binding site which causes an increase in the binding site volume, contributing further to the poor binding free energy of epinephrine to this variant (Fig. 3a). The hydration around the residue is increased marginally (Supplementary Fig. S2). Taken together these results predict that the mutation Met82Lys is detrimental to the binding of epinephrine.

### nsSNPs at the intracellular region lead to differences in G-protein coupling

**Figure 7:**
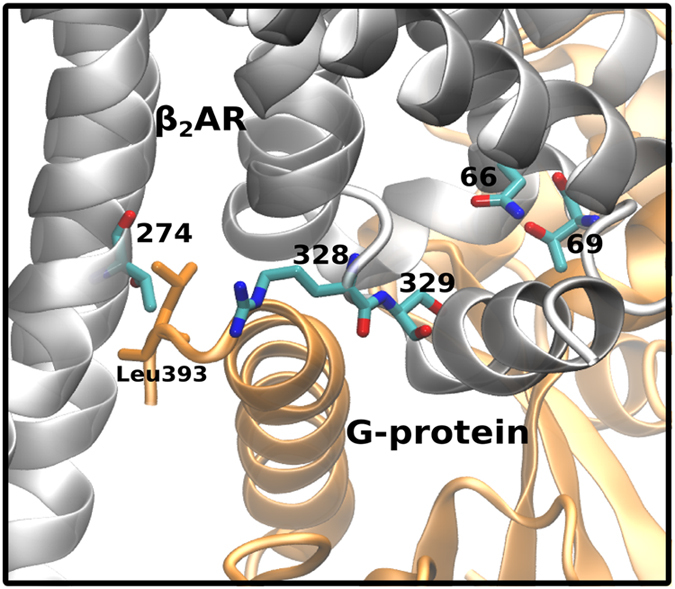
A representation of the G-protein binding site. A schematic representation of the G-protein bound structure of the receptor (3SN6) with the variant residues rendered in licorice. The receptor is coloured grey and the G-protein is coloured orange. The residue Leu 393 from the G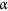 domain of G-protein that interacts with Thr274 of β_2_AR is rendered in licorice.

The variants, Thr66Met, Asn69Ser, Thr274Met, Arg328Gln and Ser329Ile are located towards the intracellular region. As discussed earlier these residues cluster in the vicinity of the G-protein as observed in the crystal structure of the ternary complex^19^ (Fig. 7). The crystal structure of the G-protein coupled to β_2_AR reveals that Thr274 interacts with Leu393 of the G_αs_ protein^19^. We therefore investigated changes in the intracellular TM helix positions caused by the variations in the inactive state of the receptor. A few variants induced minor helical rearrangements (Supplementary Fig. S5 to S8). The inactive state of the receptor thus displays minor structural differences.

To probe the effect of altered protein-protein interactions of the active state of β_2_AR with the G_αs_ protein induced by the variants, the complete β_2_AR (wild type and intracellular variants) was modelled with the G_αs_ based on the crystal structure (PDB ID: 3SN6). Further, binding free energies between the receptor and G_αs_ were calculated using MM-GBSA as described in the Methods Section. The binding free energies are reported in Supplementary Table 2. The wild type receptor displayed the most favourable binding free energy. The variants Thr66Met, Asn69Ser and Thr274Met displayed the least favourable binding free energies, while the variant Ser329Ile was similar to that of the wild type. Based on the less favourable binding free energies of the active conformation and the helical repositioning in the inactive conformation, we predict that the variants, Thr66Met, Asn69Ser and Thr274Met would have detrimental effects on G-protein coupling.

## Discussion and Conclusions

Next generation sequencing technology has enabled the quick and affordable sequencing of complete genomes. This has given rise to large scale genome sequencing projects such as the 1000 genome project and more recently the U.K. 100,000 Genomes Project and the U.S. “Precision medicine initiative” that plans to sequence the genomes of a million individuals. The sequencing of complete genomes has revealed inter-individual variability in terms of SNPs and other structural variations that has been correlated to disease susceptibility and drug response. The β_2_AR is an important GPCR that is a drug target in asthma and an off-target for cardiovascular drugs. The study of inter-individual variability of β_2_AR at the atomic level is crucial to understand the effect of variations on the structure and function. In this work we identified nine nsSNPs of the β_2_AR, classified as deleterious by a consensus prediction tool, and characterized their structural differences using molecular dynamics simulations. Based on our results we are able to classify the variants as ranging from activating to damaging and enable us to prioritise variants for experimental studies.

Surprisingly, the most well studied mutations of β_2_AR, Gly16Arg, Glu27Gln and Thr164Ile that have been correlated to a differential response to asthma drugs are missed by the consensus predictor used in our study. Although PredictSNP is described to be one of the most robust prediction tools, its accuracy has been shown to range between 72 to 78% for various datasets and could be one of the reasons for the discrepancy noted above^34^. The variants at the 16^th^ and 27^th^ position are present on the N-terminal loop that is highly variable in the GPCRs and hence could be another possible reason that the tool assigned low importance to them. In a separate study we have addressed the differential dynamics of loop positioning in the Gly16Arg leading to a differential response to albuterol^42^. Thr164 is present on TM helix 4 and is replaced by an aliphatic residue, isoleucine which would seem to be a favourable mutation in a TM region to sequence and structure based tools. Molecular insights into Thr164Ile are important and are being currently pursued by our group. Highly variable and structurally disordered regions such as the N-terminal loop and the intracellular loop 3 (ICL3) are known to be important in GPCR function^15^,^43^. However, mutations in these regions are unlikely to be classified as deleterious by the current tools. This necessitates the development of a GPCR specific predictor in the light of it being the most important class of drug targets.

The most interesting variant identified in our study was the Phe290Ser that appears to be an activating mutation. This mutation was predicted to be deleterious by PredictSNP which predicts a mutation to diverge from the wildtype behaviour but cannot distinguish whether the altered behaviour deprecates or improves its function. The activation mechanism of class A GPCRs is hypothesized to involve the flipping of the sidechain of Trp^6.48^ from a *gauche* (inactive) to a *trans* (active) state. This flip has been observed in spectroscopic studies and molecular dynamic simulations of the rhodopsin and adenosine_2A_ receptor^28^,^29^,^38^,^39^. Additionally, mutagenesis and Monte Carlo simulations have predicted the presence of this rotamer transition in β_2_AR^44^. Since this flip has never been observed in crystal structures of β_2_AR its role in activation has been recently questioned^45^. Interestingly, we observed a transient flip of Trp^6.48^ in all three sets of the variant simulations that was absent in the simulations of the wildtype receptor and the negative controls. The increased dynamics of the crucial Trp^6.48^, one of the first steps in activation, could facilitate receptor activation kinetics.

Hydrophobic mismatch has been observed in the wildtype receptor that leads to local membrane thickening and thinning in its vicinity, in particular thickening around the groove between TM helix 1 and 7^41^,^46^,^47^. Association of β_2_AR and related GPCRs into dimers and higher order oligomers has been demonstrated to occur at these sites of membrane perturbations^41^,^48^. An interesting set of mutations namely Ala85Gln, Thr274Met and Gly320Asp, were identified in our study in which phospholipid association with the receptor was observed causing the head group of the lipid molecule to be pulled toward to the receptor. This led to large membrane perturbations that varied from the wildtype receptor. These perturbations of the variants propagate through the membrane causing widespread membrane thinning. Additionally, the membrane thickening at TM helix 1 and 7 is reduced. As reported above, a series of simulation studies have linked GPCR association to local lipid perturbations^41^,^48^. In addition, membrane thickness has been related to receptor conformational changes as well as activation in rhodopsin^49^. Thus, these predicted membrane perturbations have implications in altered β_2_AR association in the Ala85Gln, Thr274Met and Gly320Asp variants.

Further the mutation, Gly82Lys appears to be unfavourable for ligand binding. In particular, the introduction of a positive charge in the binding site alters the negative electrostatic potential that facilitates epinephrine binding. The lysine residue also disturbs the aromatic core at the bottom of the binding site leading to a larger volume. As a consequence, this variant shows a much reduced binding free energy for epinephrine as compared to the wildtype receptor. In such a scenario, increased epinephrine concentration would be required for optimal receptor activity.

The variant Thr274Met disrupts a critical contact with G-protein. The loss of this contact assumes added significance due to the fact that there are surprisingly very limited contacts observed between the β_2_AR and the G_αs_ protein. A few of the intracellular variants induce small rearrangements of TM helices in their vicinity. Further, in the active state the variants Thr66Met, Asn69Ser and Thr274Met have less favourable binding free energy with G_αs_ as compared to wild type receptor. In all, these local rearrangements and reduced G_αs_ binding would have consequences on downstream signalling. These variants would thus also appear to be detrimental.

State of art simulation methods that encompass several length and time-scales are increasingly being used to analyse various facets of GPCR structure and function^50^. In this work we report local structural perturbations of β_2_AR variants occurring at ns timescale. At longer timescales global mechanisms such as receptor activation can be observed^23^,^24^ The key observations for the variants reported in this work are robust at an extended sampling of 0.6 μs (Supplementary Fig. S9). An important hallmark of β_2_AR is its ability to bind its natural ligand R-epinephrine as well as several synthetic agonists and antagonists. In this study we employed docking and MM-GBSA methods to predict the interactions of the variants with R-epinephrine. However to test the robustness of the method we calculated the binding free energies for seven ligands with the wild type receptor and compared them to experimental values^37^ (Supplementary Fig. S10). We observe a high correlation (0.86) between the experimental and computational energies improving the confidence of our predictions for the variants. This finding is in line with that of Vilar *et al*. where they suggest that MM-GBSA is the method of choice for prediction of β_2_AR ligand affinities. However the general applicability of the method has been questioned^51^. MM-GBSA has also been used to quantify the affinity between complexes of proteins, wherein a good correlation between experimental and computational values was observed^52^,^53^. We used the method to calculate the binding energetics of the interaction between variant receptors and the G_αs_ protein. However, experimental validation for this system is lacking. Taken together, in this work we use computational tools to design a framework that could bridge genome based predictions to detailed biophysical studies based on structural insights.

In conclusion, this work highlights the local structural reorganization induced by nine deleterious nsSNPs of β_2_AR enabling us to understand their functional implications. In particular we suggest the Phe290Ser mutant as being activating, while the rest except Ser329Ile as damaging. Specifically, ligand binding is adversely affected in the Met82Lys variant. Large membrane perturbations are observed in a few variants with implication in receptor organization. G-protein coupling is suggested to be affected in several variants at the intracellular region. This work is an important step in characterising the inter-individual variability in β_2_AR at the structural level, helping in prioritising variants that must be experimentally tested.

## Methods

### Compilation and prediction of deleterious nsSNPs

nsSNPs for human β_2_AR were compiled from the dbSNP (http://www.ncbi.nlm.nih.gov/snp)^54^. The effect of the mutation on function was predicted using the consensus deleterious SNP detector, PredictSNP^34^. PredictSNP is a web-based tool that combines six methods, MAPP^55^, Polyphen-1^56^, Polyphen-2^57^, SNAP^58^, PHD-SNP^59^ and SIFT^60^. PredictSNP compiles results from each tool and provides a comprehensive score ranging from 0–100%, indicating the level of damage the SNP might have on protein function.

### Structural characterisation and dynamics of deleterious nsSNPs

A structural model of wildtype β_2_AR was built based on its crystal structure (PDB ID: 2RH1), in which the ICL3 was modelled using the templates: bovine rhodopsin (1U19), squid rhodopsin (2ZIY) and the NKR1p-subunit (2KS9) using Modeller version 9.7^61^. Models for the individual nsSNPs were built by replacing the naturally occurring amino acid with its variant using Discovery Studio 3.5^62^. The models were embedded in a fully hydrated POPC (1-palmitoyl-2-oleoyl-sn-glycero-3-phosphocholine) bilayer using a computational tool, g_membed^63^ and molecular dynamics simulations were performed. The protein and lipid parameters were derived from the CHARMM36 force-field^64^,^65^ and the simulation was carried out using the GROMACS package version 4.5.5^66^. The TIP3P water model was used to represent water molecules. Periodic boundary conditions were applied in all directions with a semi-isotropic pressure coupling scheme with Parrinello-Rahman barostat^67^. Long range electrostatics were treated using the PME method implemented in GROMACS. The system temperature was maintained at 300 K by coupling to a Nosé-Hover thermostat^68^. The system was energy minimized using the Steepest Descent algorithm. Subsequently, 100 ps of NVT and 1 ns of NPT equilibration was carried out. The wild-type model and the nine variant models were simulated for 200 ns in triplicate totalling to 6 μs of simulation time. Further, the variant Q179E was modelled as per the procedure discussed above and was treated as a negative control, while a protonated form of Asp 320 was considered as an additional control for that mutant. Both of these simulations were performed for 200 ns.

### Analysis

All analysis was performed using standard GROMACS and VMD^69^ tools. For the RMSD calculation, the TM region was aligned to 2RH1 crystal structure. The calculation of local water was performed by calculating water molecules within 0.8 nm of the Cα atom of each variant and wildtype residues individually. The membrane thickness was calculated as the average distance between the phosphorous atoms in the headgroup of POPC bilayer using a previously developed analysis script^40^,^70^. The dihedral angle, χ^1^ of Trp 286^6.48^ is defined by the residues (N- Cα- Cβ-Cγ).

### Docking calculations

Ligand docking calculations were performed using Maestro version 9.4^71^. Protein structures representing the last frame of the simulations were prepared using the Protein preparation wizard and the ligand epinephrine was prepared in the R form using the Ligprep module. Residues that were within 0.7 nm of carazolol in the 2RH1 structure were used to define the centroid of the grid for docking in the wildtype and corresponding variants. Docking was performed using GLIDE-XP^72^ with all default parameters. The docked complexes were minimized using the local optimisation feature in Prime, and the energies were calculated using the OPLS3 force field and the GBSA continuum membrane and solvent model in Maestro. The binding free energy was calculated as the difference in free energy between the complex and the sum of free energies for the unliganded protein and ligand. The complete β_2_AR (wild type and intracellular variants) was modelled with the G_αs_ based on the crystal structure (PDB ID: 3SN6). Further, the protein-protein binding free energies were calculated similarly.

### Binding pocket volume calculation

MDpocket^73^, a tool based on Voronoi tessellation, was used for detecting pockets along the course of the simulation. Residues within 0.7 nm of carazolol in the 2RH1 structure were used to define the binding pocket. Initially, all the trajectories were superimposed based on the binding pocket and a mesh was placed in voids between the residues defining the binding pocket. All the trajectories aligned by entire length along with the grid were submitted for the final calculation.

## Additional Information

**How to cite this article**: Tandale, A. *et al.* Structural insights and functional implications of inter-individual variability in β_2_-adrenergic receptor. *Sci. Rep.* **6**, 24379; doi: 10.1038/srep24379 (2016).

## Supplementary Material

Supplementary Information

## Figures and Tables

**Table 1:** List of deleterious nsSNPs predicted by PredictSNP.

SNP position	PredictSNP (Confidence)	Meta-SNP (Confidence)	PON-P2 (Probability)
Thr66Met	Deleterious (87%)	Disease (0.71)	Pathogenic (0.85)
Asn69Ser	Deleterious (87%)	Disease (0.82)	Pathogenic (0.87)
Met82Lys	Deleterious (87%)	Disease (0.78)	Pathogenic (0.88)
Ala85Gln	Deleterious (76%)	Disease (0.78)	Pathogenic (0.85)
Thr274Met	Deleterious (87%)	Disease (0.83)	Pathogenic (0.83)
Phe290Ser	Deleterious (87%)	Disease (0.83)	Pathogenic (0.81)
Gly320Asp	Deleterious (87%)	Disease (0.76)	Pathogenic (0.80)
Arg328Gln	Deleterious (87%)	Neutral (0.47)	Pathogenic (0.88)
Ser329Ile	Deleterious (87%)	Disease (0.76)	Pathogenic (0.88)
