# Supplementary material for: Structural insights and functional implications of inter-individual variability in β2-adrenergic receptor
Source: Sci Rep. 2016 Apr 14;6:24379. doi: 10.1038/srep24379 (PMC4830965; doi:10.1038/srep24379)
Supplement: Supplementary Information [file srep24379-s1.pdf]

Supplementary Information

Structural insights and functional implications of inter-individual variability in β_2-adrenergic receptor

Aditi Tandale[1], Manali Joshi[2]* and Durba Sengupta[1]*

1. CSIR-National Chemical Laboratory, Dr. Homi Bhabha Road, Pune 411008, India

2. Bioinformatics Centre, S.P. Pune University, Ganeshkhind Road, Pune 411007, India

Supplementary Table S1: List of nsSNPs of β_2AR obtained from dbSNP

Sr. No.	rsID	Nucleotide Polymorphism	Protein Position	Amino Acid Polymorphism
1	rs151019714	A/G	1	M [Met] ⇒ V [Val]
2	rs148459047	A/C	4	P [Pro] ⇒ H [His]
3	rs144618094	A/G	15	N [Asn] ⇒ D [Asp]
4	rs33973603	A/G	15	N [Asn] ⇒ S [Ser]
5	rs35892629	A/G	27	Q [Gln] ⇒ K [Lys]
6	rs373763314	C/T	66	T [Thr] ⇒ M [Met]
7	rs201257377	A/G	69	N [Asn] ⇒ S [Ser]
8	rs371307481	C/G	73	T [Thr] ⇒ S [Ser]
9	rs140420820	A/T	82	M [Met] ⇒ K [Lys]
10	rs113509650	A/G	85	A [Ala] ⇒ Q [Gln]
11	rs145233416	C/T	89	F [Phe] ⇒ L [Leu]
12	rs201777403	A/G	92	A [Ala] ⇒ T [Thr]
13	rs373098969	C/G	95	L [Leu] ⇒ V [Val]
14	rs149199162	G/T	128	A [Ala] ⇒ S [Ser]
15	rs1800888	C/T	164	T [Thr] ⇒ I [Ile]
16	rs147102529	A/C	178	H [His] ⇒ Q [Gln]
17	rs148196791	C/G	179	Q [Gln] ⇒ E [Glu]
18	rs200042760	A/G	187	N [Asn] ⇒ S [Ser]
19	rs370652553	A/G	218	V [Val] ⇒ I [Ile]
20	rs3729943	C/G	220	S [Ser] ⇒ C [Cys]
21	rs150750214	C/G	224	Q [Gln] ⇒ E [Glu]
22	rs200987369	G/T	228	R [Arg] ⇒ S [Ser]
23	rs143912954	C/T	233	I [Ile] ⇒ T [Thr]
24	rs41320345	C/T	240	F [Phe] ⇒ L [Leu]
25	rs372301487	C/G	244	N [Asn] ⇒ K [Lys]
26	rs41358746	G/T	247	Q [Gln] ⇒ H [His]
27	rs56100672	A/G	257	G [Gly] ⇒ R [Arg]
28	rs201323763	A/G	259	R [Arg] ⇒ H [His]
29	rs374596758	C/T	274	T [Thr] ⇒ M [Met]
30	rs201318801	C/T	290	F [Phe] ⇒ S [Ser]
31	rs375254430	A/G	292	V [Val] ⇒ I [Ile]
32	rs369459230	A/G	296	H [His] ⇒ R [Arg]
33	rs371880712	A/G	297	V [Val] ⇒ M [Met]
34	rs377115303	C/T	304	R [Arg] ⇒ C [Cys]
35	rs142595411	A/G	320	G [Gly] ⇒ D [Asp]
36	rs149939087	A/G	328	R [Arg] ⇒ Q [Gln]
37	rs201644897	G/T	329	S [Ser] ⇒ I [Ile]
38	rs374400618	A/C	370	Q [Gln] ⇒ K [Lys]
39	rs140258602	C/G	383	G [Gly] ⇒ R [Arg]
40	rs373075228	G/T	388	V [Val] ⇒ G [Gly]
41	rs374211656	A/G	392	G [Gly] ⇒ S [Ser]

Supplementary Table S2: Binding free energy between the variant receptors and $G_{\alpha s}$ calculated by MM-GBSA method

Variant	Binding Free Energy (kcal/mol)
WT	-470.4
Thr66Met	-257.1
Asn69Ser	-237.0
Thr274Met	-250.6
Arg328Gln	-340.3
Ser329Ile	-459.3

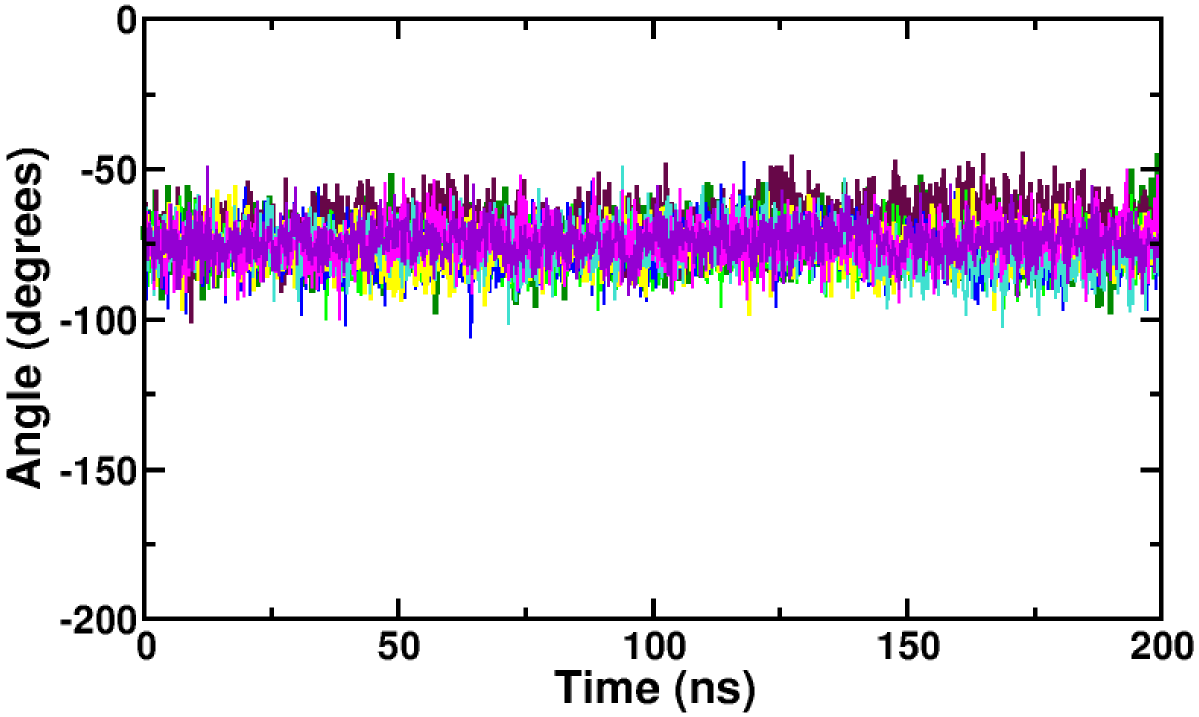

Supplementary Figure S1: A plot of the χ^1 angle of Trp286 along the course of the simulation for all variants in triplicates except Phe290Ser.

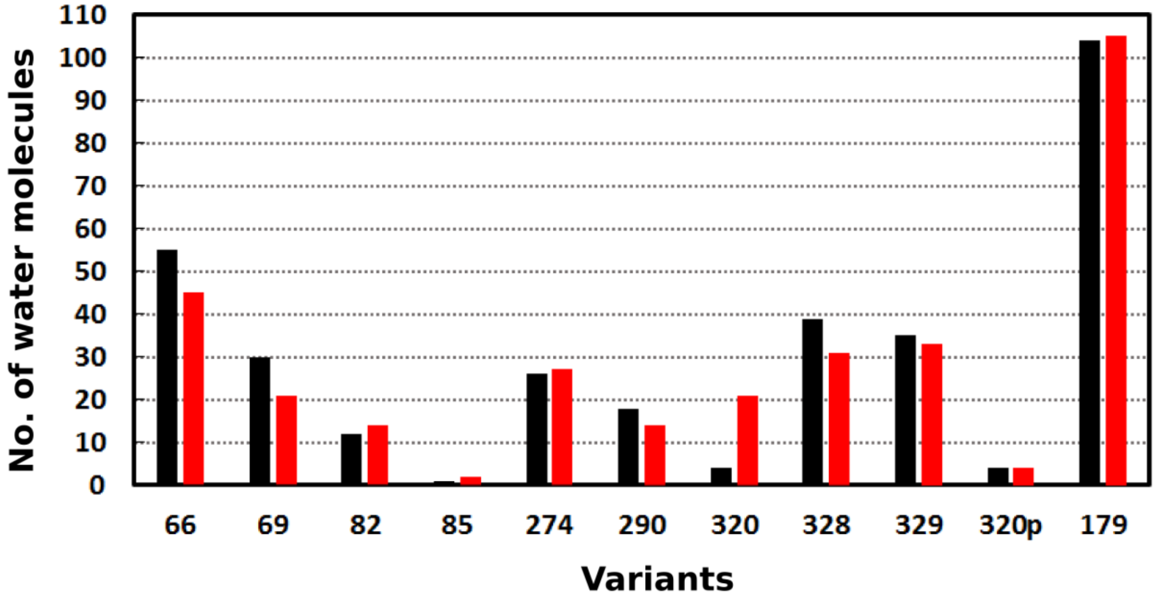

Supplementary Figure S2: Number of water molecules in the vicinity of the variant and the wildtype residues (within 0.8 nm of the Cα of the residue). Black bars represent the average values from the triplicate simulations of the wild type and the red represents the average values from the triplicate simulations of the variant receptors.

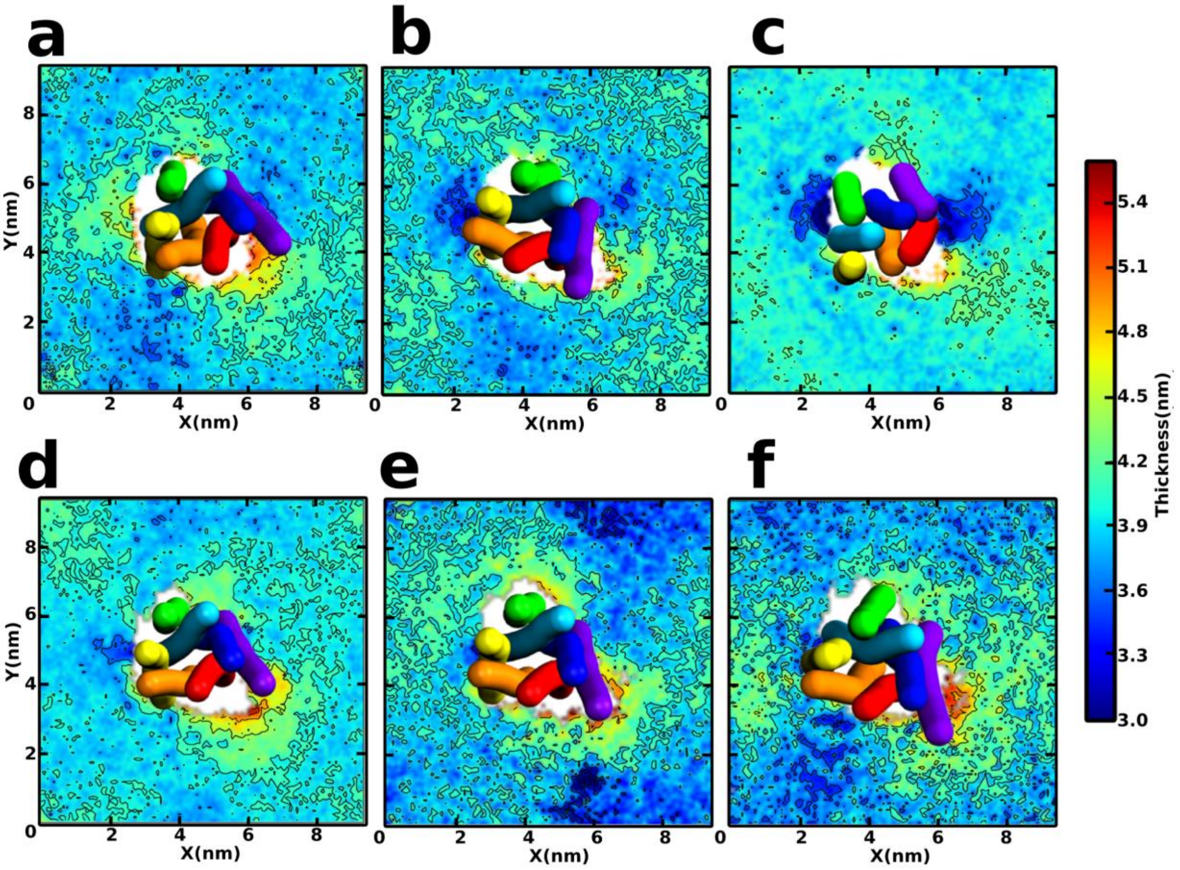

Supplementary Figure S3: Average bilayer thickness profile over simulations sets of (a) Ala85Gln (b) Thr274Met (c) Gly320Asp (d) Wild type receptor (e) Negative control and (f) Protonated Gly320Asp. The scale bar for the membrane thickness is given at the right side of the panel. The receptor structure is superimposed on the thickness profiles and the transmembrane helices are coloured as in Figure 1 of the manuscript.

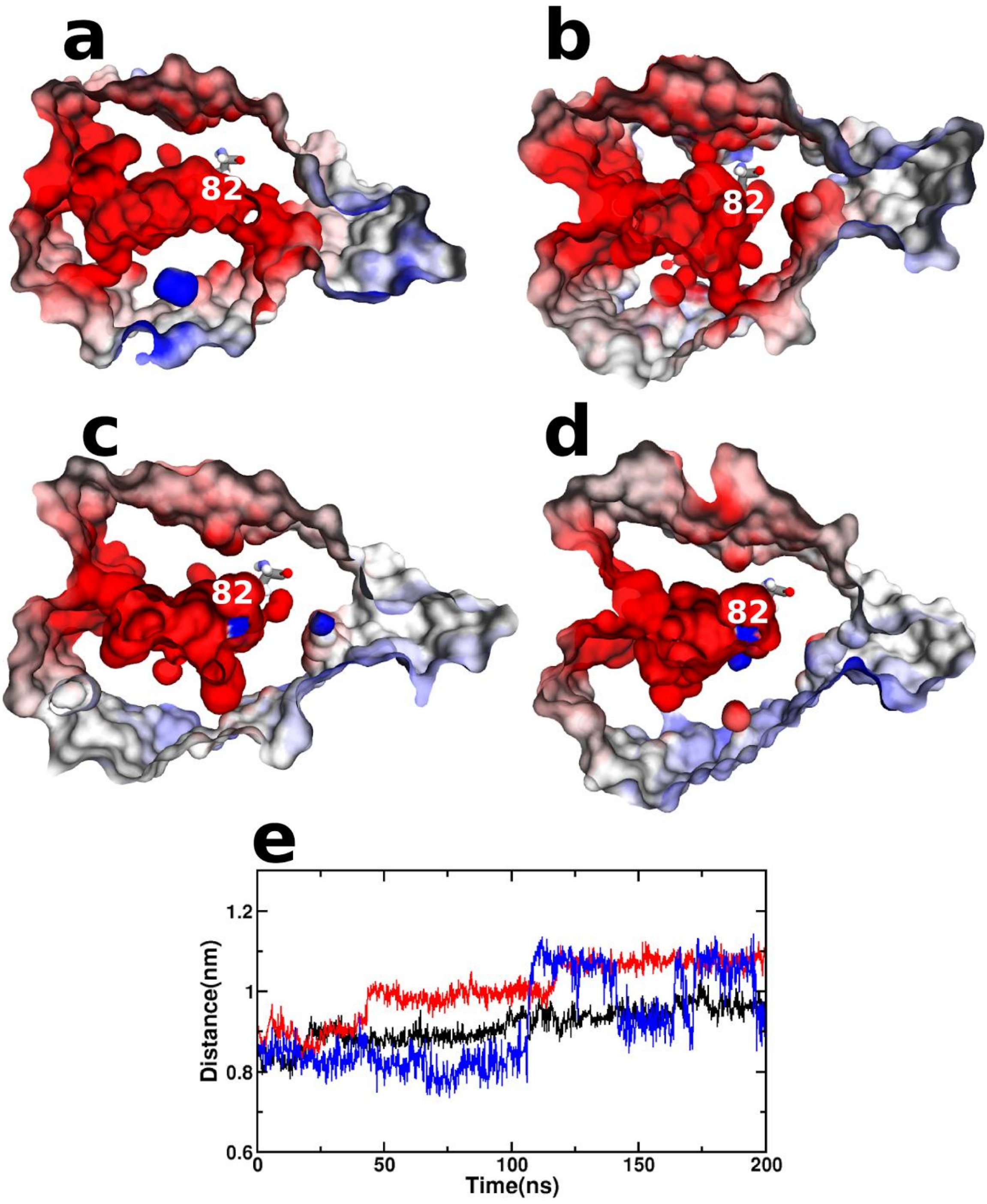

Supplementary Figure S4: Electrostatic potential maps of the binding site of (a) wild type set 2 (b) wildtype set 3 (c) Met82Lys variant set 2 (d) Met82Lys variant set 3. (e) A plot of the average distance of the Cα atoms of residues 286 and 316. Black line represents the average value for wildtype over three sets; the red line represents average value for the variant Met82Lys over three sets while the blue line represents the value for the negative control.

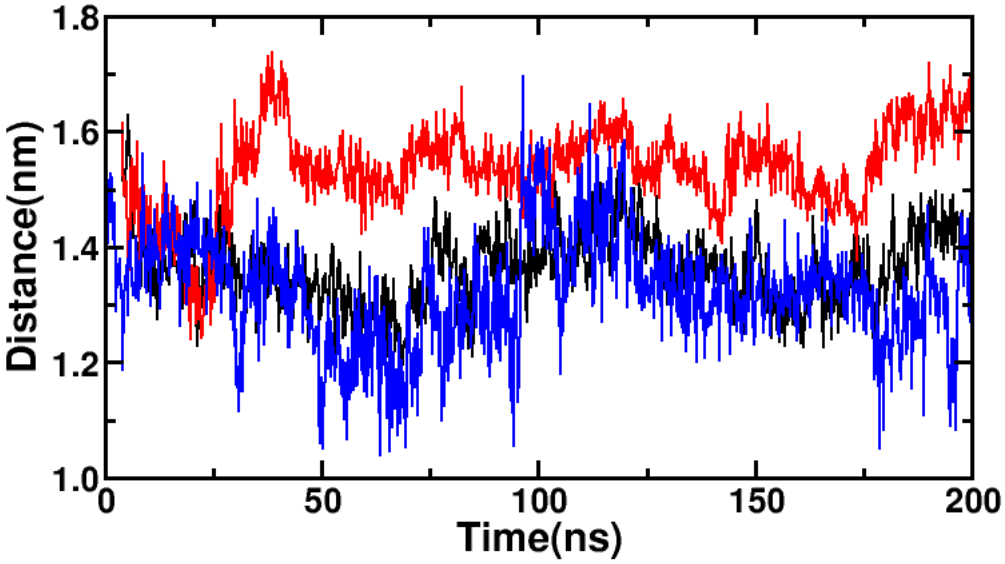

Supplementary Figure S5: Distance between Cα atoms of residue 66 on TM helix 2 and residue 267 on TM helix 6. Black line represents the average value for wildtype over three sets, the red line represents average value for the variant Thr66Met over three sets while the blue line represents the value for the negative control.

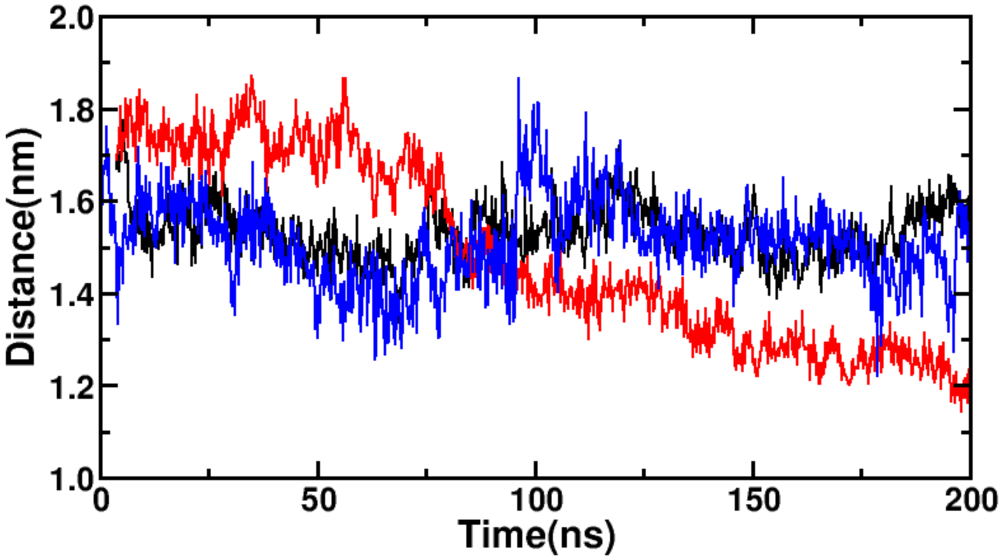

Supplementary Figure S6: Distance between Cα atoms of residues 67 (TM helix 2) and 267 (TM helix 6). Black line represents the average value for wildtype over three sets, the red line represents average value for the variant Asn69Ser over three sets while the blue line represents the value for the negative control.

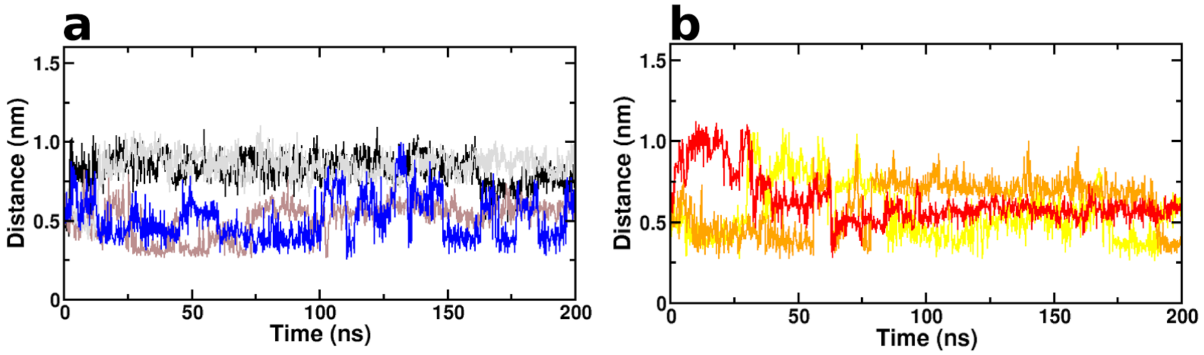

Supplementary Figure S7: Distance between sidechain atoms of residues 328 (TM helix 7) and 333 (helix 8) for (a) Wild type (black, grey, brown) and negative control (blue) and (b) variant Arg328Gln (red, yellow, orange).

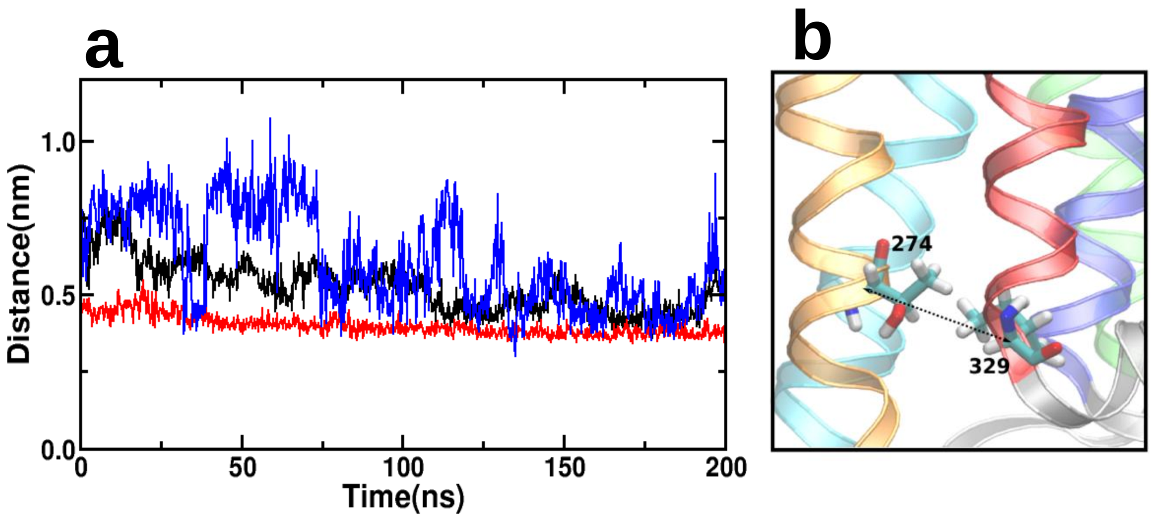

Supplementary Figure S8: (a) Distance between sidechain atoms of residues 274 (TM helix 6) and 329 (TM helix 7). Black line represents the average value for wildtype over three sets, the red line represents average value for the variant Ser329Ile over three sets while the blue line represents the value for the negative control. (b) The schematic representation of the variant Ser329Ile with the residues 274 and 329 represented in licorice.

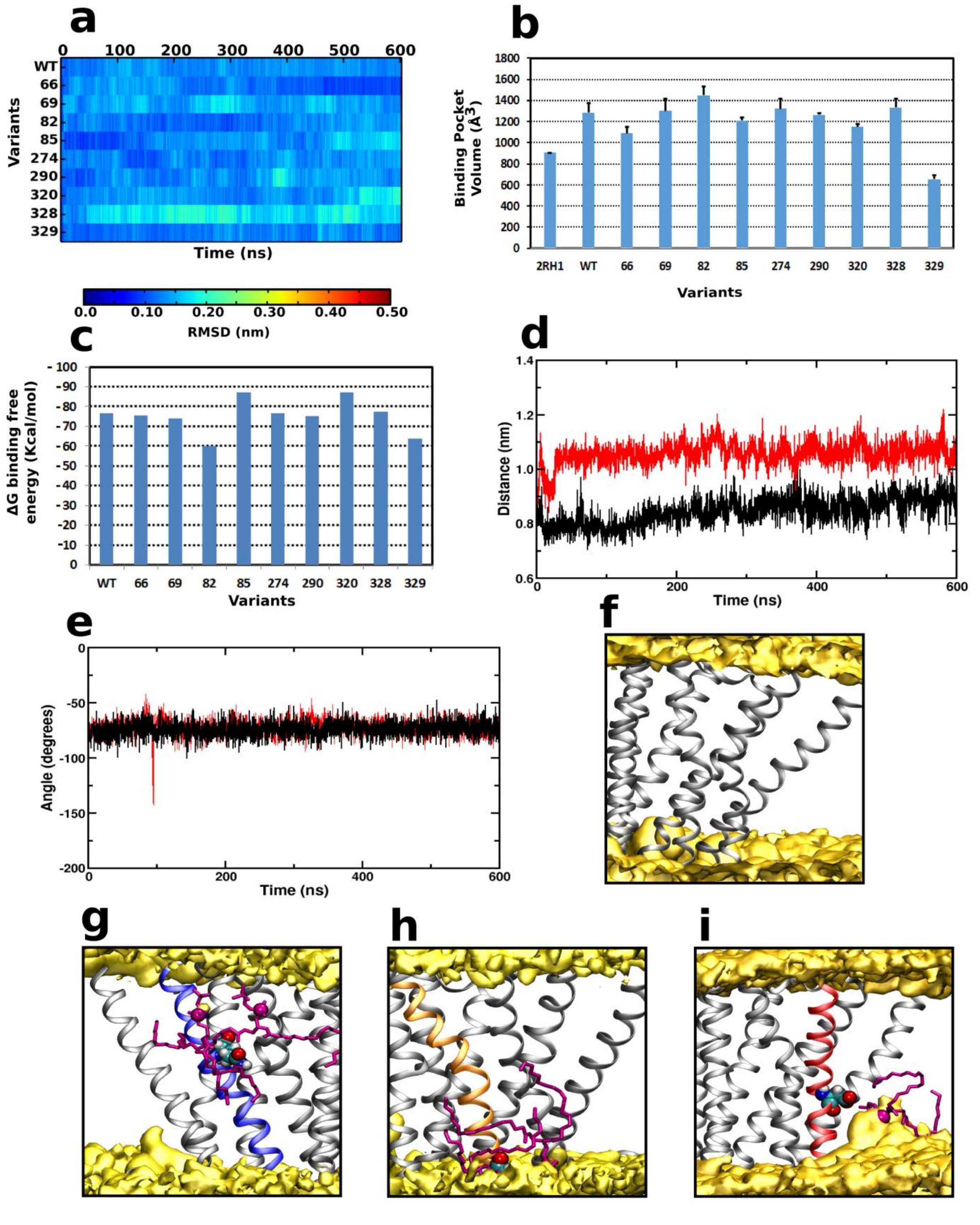

Supplementary Figure S9: Extended analysis of set 1 of wild type and nine variant simulations (a) RMSD of the transmembrane helices. (b) Binding pocket volume averaged over the simulation. (c) The binding free energy of R-epinephrine for the variants at 600ns. (d) A plot of the distance of the Cα atoms of residues 286 and 316. Black line represents the wildtype and the red line represents the variant Met82Lys. (e) A plot of the dihedral angle, $\chi 1$ of Trp286 along the course of the simulation. Schematic representation of average lipid head group (phosphate atom) position of POPC in the vicinity of the receptor for (f) wildtype (g) Ala85Gln, (h) Thr274Met (i) Gly320Asp

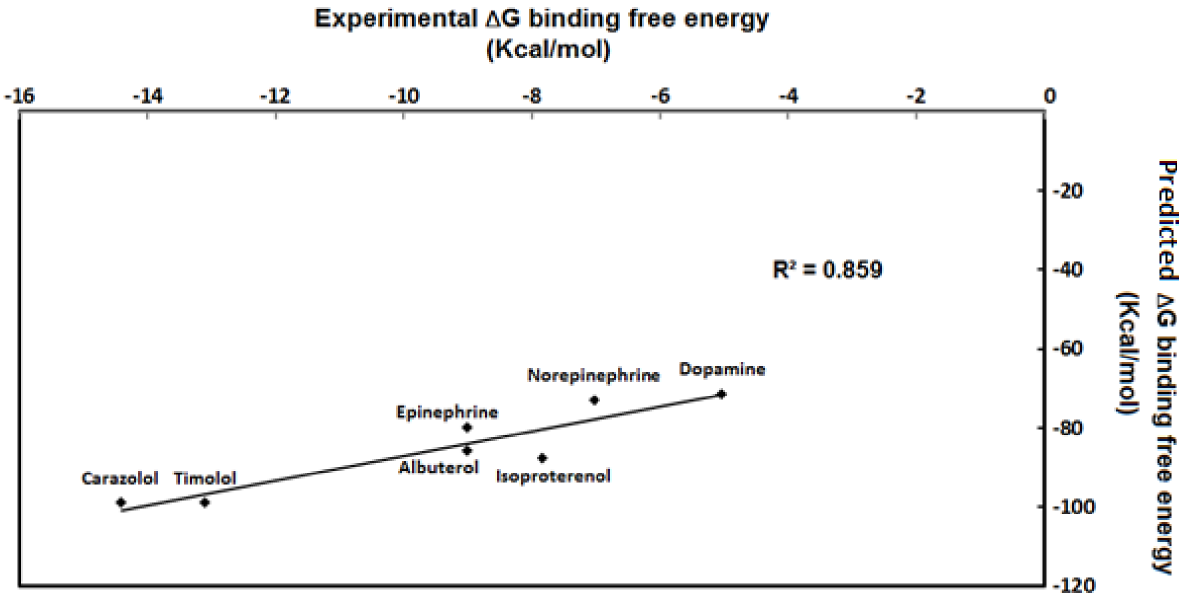

Supplementary Figure S10: Correlation plot between the experimental and predicted binding free energies for seven β_2AR ligands. The ligands considered were epinephrine, norepinephrine, carazolol, albuterol, timolol, isoproterenol, dopamine. The experimental values were taken from Vilar *et al.* (Ref. 37).
